# Changes in patient-reported outcomes during admission to a South African psychiatric facility

**DOI:** 10.4102/sajpsychiatry.v30i0.2258

**Published:** 2024-09-30

**Authors:** Lerato Motshudi, Cherie-Dee Hann, Marilee Kloppers, Thierry Luhandjula, Tiro Phalatse, Damien Pretorius, Dianne Smith, Manuela Smith, Marius van der Westhuizen, Reitze N. Rodseth

**Affiliations:** 1 Netcare Ltd, Johannesburg, South Africa; 2 Department of Anesthesiology and Critical Care, Nelson R. Mandela School of Medicine, University of KwaZulu-Natal, Durban, South Africa

**Keywords:** Patient-reported outcome, PROMS, depression, PHQ-9, depression score

## Abstract

**Background:**

Patient-reported outcome measures (PROMs) are used as part of clinical practice to determine the impact of the condition and treatment interventions on a patient’s health and quality of life. The Patient Health Questionnaire-9 (PHQ-9) is a self-administered diagnostic tool that has been widely adopted for the detection and monitoring of depression.

**Aim:**

This analysis reports the change in PHQ-9 scores from admission to discharge in patients admitted for depression to a South African acute psychiatric facility and aims to quantify the treatment effect of the admission using the PHQ-9 as the measurement tool.

**Setting:**

South African acute psychiatric facility.

**Methods:**

This was a retrospective observational study of all patients admitted to Netcare Akeso acute psychiatric facilities from 01 January 2018 to 31 October 2022. Patients were included if they were ≥ 18 years of age, admitted with a primary International Classification of Disease (ICD)-10 code for depression (i.e. F32–F33) and fully completed both an admission and discharge PHQ-9 questionnaire. We excluded facilities focusing only on the treatment of patients with specialised conditions such as addiction or eating disorders.

**Results:**

This analysis included 13 308 patients admitted for depression at 10 different facilities. The median PHQ-9 score on admission was 19 (interquartile range [IQR] 14–23) and 5 (IQR 2–11) on discharge, with a median change of -12 (IQR -5 to -18). A minimal clinically important difference was seen in 87.6% patients (*n* = 10 091/11 515); a treatment effect was seen in 74.5% of patients and a clinically significant improvement was seen in 72.1% of patients.

**Conclusion:**

With the average patient reporting a four-fold reduction in the severity of their depression scores, PROMs provide a critical patient-centred window into the benefit that an inpatient admission has on those suffering with depression.

**Contribution:**

These changes are consistent with those seen internationally and provide a baseline for understanding the treatment efficacy of an inpatient admission for the treatment of depression.

## Introduction

Patient-reported outcome measures (PROMs) are tools or measurement instruments that are used to compile and report patient reported outcomes (PRO). An example of a PRO is a patient’s rating of their pain, and an example of a PROM would be a pain scale.

Patient-reported outcome measures are used as part of clinical practise to determine the impact of treatment interventions on a patient’s health and quality of life and have become key in developing and improving patient-centred care.^1^ Studies have suggested that their use enriches communication between patients and healthcare providers and may hasten patient improvement.^2,3,4^

The Patient Health Questionnaire-9 (PHQ-9) is a self-administered diagnostic tool that has been widely adopted for detection and monitoring of depression and is commonly used as a PROM.^5,6^ Since 2018, the questionnaire has been progressively introduced into the Akeso group of acute care psychiatric facilities, part of Netcare Limited ‘Netcare’, a South African private healthcare group. This group of psychiatric facilities includes private sector hospitals, dedicated to psychiatric care. They admit an average of 18 000 mental health patients per year, with over 60% of these presenting with depression. The various facilities are located in different parts of the country and service mostly urban and semi-urban populations.

There are a variety of ways to understand this score and its changes. The first is to quantify the smallest change in the PHQ-9 score that the patient would consider to be significant. This is called the minimal clinically important difference (MCID). The MCID is generally determined within a specific patient population (e.g. cardiac surgery, chronic obstructive pulmonary disease, rheumatology patients), but previous studies have reported MCID thresholds in patients with depression. These have been defined as a reduction in PHQ-9 score by ≥ 5 for patients starting with a score of 5 or more,^7^ an average change of 3.4 points^8^ or an average change of 3.5 or a 20% improvement in scores.^9^ The second way is to identify patients who show a treatment response, defined as a 50% reduction in PHQ-9 score for patients starting with a score of ≥ 5^10^ and the third is to identify those patients who show a clinically significant improvement – defined as a 50% PHQ-9 reduction from admission score and a discharge PHQ-9 score < 10 for patients starting with a score ≥ 10.^5^

While most depressive patients can be managed as outpatients, inpatient care may be indicated for severe depression or cases associated with suicidal behaviour, psychotic symptoms, catatonic symptoms, poor physical health or a lack of social support.^11^ Treatments provided during inpatient admission may include antidepressants, individual and group therapy and in some cases electroconvulsive therapy.^12^ The majority of South Africa’s population is reliant on the public healthcare system that is understaffed and underfunded, with a focus on specialist psychiatric hospitals.^13,14^

In this analysis, we will report the change in PHQ-9 scores from admission to discharge, in patients admitted for depression. The analysis will quantify the treatment effect of the admission in terms of the MCID, treatment response and clinically significant improvement.

## Research methods and design

This was a retrospective observational study of all patients admitted to the acute psychiatric facilities from 01 January 2018 to 31 October 2022. On admission, all patients are given a paper copy of the PHQ-9 assessment to complete and again at discharge. The forms are scanned and submitted to a central point where the data are captured.

Patients were included if they were ≥ 18 years of age, admitted with a primary ICD-10 code for depression (i.e. F32–F33) and fully completed both an admission and discharge PHQ-9 questionnaire in English. We excluded facilities focusing only on the treatment of patients with specialised conditions such as addiction or eating disorders. There were no comorbid diagnoses that were considered as exclusion criteria.

From the included patient data, we determined the median admission and discharge PHQ-9 score. We then quantified the patient’s response to the admission using these previously defined treatment thresholds:

- Minimal clinically important difference – a reduction in PHQ-9 score by ≥ 5 for patients starting with a score of ≥ 5,^7^
- Treatment response – a 50% reduction in PHQ-9 score for patients starting with a score of ≥ 5^10^ and
- Clinically significant improvement: 50% PHQ-9 reduction from admission score and a discharge PHQ-9 score < 10 for patients starting with a score ≥ 10.^5^

We further explored the characteristics of those patients whose PHQ9 score worsened during the admission period.

The PHQ-9 questionnaire has been extensively tested and validated and is available in over 30 languages. The questionnaire has nine questions, and for each question, there are four possible responses, each with an associated value: Not at all (0), Several days (1), More than half the days (2), Nearly every day (3). The questions are as follows:

Over the last 2 weeks, how often have you been bothered by any of the following problems?

- Little interest or pleasure in doing things.
- Feeling down, depressed or hopeless.
- Trouble falling or staying asleep or sleeping too much.
- Feeling tired or having little energy.
- Poor appetite or overeating.
- Feeling bad about yourself – or that you are a failure or have let yourself or your family down.
- Trouble concentrating on things, such as reading the newspaper or watching television.
- Moving or speaking so slowly that other people could have noticed? Or the opposite – being so fidgety or restless that you have been moving around a lot more than usual.
- Thoughts that you would be better off dead or of hurting yourself in some way.

Analysis of these data was conducted using Excel (Microsoft Corporation 2022) and R version 3.5.3 (R Core Team 2017; R Foundation for Statistical Computing, Vienna, Austria). Continuous variables were summarised as mean and standard deviation (SD) for normally distributed data or median and interquartile range (IQR) for non-normally distributed data. *T*-tests or Mann–Whitney tests were used for continuous and Chi-square with Yates correction chi-square tests for categorical variables. Statistical significance was set at *p* < 0.05.

On admission, patients provided written consent for their data to be used in conducting research and analysis. Ethics approval for this analysis was obtained from the Pharma-ethics research ethics committee (Reference Number: 230925851).

### Ethical considerations

Ethical clearance to conduct this study was obtained from the Pharma-Ethics Independent Research Ethics Committee (No. 230925851).

## Results

We identified 50 235 PHQ-9 records, of which 18 928 adult patients completed both an admission and discharge PHQ-9 questionnaire. Of these, 70% (*n* = 13 308) were admitted for depression and were included in this analysis. Patients were admitted at 10 different facilities across the country. Most patients were female 66% (*n* = 8772) with a median age of 36 years (IQR 28–46). For 61% (*n* = 7639) of patients, it was their first admission to a psychiatric hospital and their median length of stay was 15 days (IQR 10–20).

The median PHQ-9 score on admission was 19 (IQR 14 – 23) and 5 (IQR 2–11) on discharge, with a median change of -12 (IQR -5 to -18). An MCID was achieved in 87.6% patients (*n* = 10 091/11 515), a treatment effect was seen in 74.5% of patients (*n* = 8582/11 515) and a clinically significant improvement was seen in 72.1% of patients (*n* = 7758/10 766).

Within the study population, 12.2% of patients (*n* = 1627) reported a worsening in their PHQ-9 with a median increase of 5 (IQR 2 – 9). Those who worsened were significantly older with a median age of 37 years (IQR 29–47) versus 36 years (IQR 28–45; *p* < 0.001), had a significantly longer length of stay 15 days (IQR 10-20) versus 14 days (IQR 9-19; *p* < 0.001), had a lower proportion of females (56.2% vs 67.3%; *p* < 0.001) and a higher proportion of first time admissions (63.8% vs 61.2%; *p* = 0.045).

## Discussion

Patient-reported outcome measures provide a critical patient-centred window into the benefit that an admission into a psychiatric facility has on those suffering with depression. On average, patients report a four-fold reduction in the severity of their depression scores; 88% show a change that meets or exceeds what would be considered a minimal clinically important improvement in their severity; 75% report a treatment response equal to a 50% reduction in their severity and 72% report a clinically significant treatment response.

These results compare well with international literature. A study comprising 1023 US patients examining PHQ-9 score changes during the management of depressive in-patients reported mean admission scores of 14.5 (SD 6.6) and discharge scores of 9.9 (SD 5.9), with a post-treatment change of -13.2.^15^ The patients received cognitive behavioural therapy, pharmacotherapy and aftercare planning as part of their treatment program. In a second study comprising 27 991 patients drawn from 41 different US facilities participating in routine outcomes monitoring and benchmarking, the average admission score was 14.6 and average discharge score was 4, and patients showed an average decrease of -9.7.^16^ In a third analysis, examining three cohorts of medical outpatients (*n* = 167), the change in PHQ-9 score ranged from -10.8 (SD 5.7) to – 5.14 (SD 4.9).^7^

Of interest is the subset of patients who reported a worsening in their PHQ-9. These patients were on average older, male and were more likely to be admitted for the first time. Additional research is required to further understand this patient population.

For selected patients, inpatient treatment for depression seems to have some advantages over treatment as usual. In a study of 280 patients with chronic depression, inpatient admission for short-term psychotherapy was superior to controls.^17^ Similarly, a meta-analysis of 14 studies with 1080 major depressive patients, found that inpatient psychotherapy, as compared to different control conditions resulted in a statistically significant benefit that persisted over 12 months of follow-up.^18^ However, as inpatient treatment is resource intensive, programs that provide intensive outpatient support may be an alternative.^19^

## Conclusion

This is one of the first studies in South Africa to explore the utility of PROMs in monitoring patients being treated for depression. The standardised methodology of the data collection, together with the large sample size, adds to the reliability of these results. The study is limited in that it only examined patients who completed PHQ-9 surveys at admission and discharge. Patients with more severe depression may not have completed a PHQ-9 on admission or discharge and would therefore not be included in this analysis. We did not derive a study-specific MCID but made use of previously published thresholds. This presents itself as a subject for future investigation. Finally, the analysis is further limited by its retrospective use of administratively collected data and a lack of a control group.^20,21^

With the average patient reporting a four-fold reduction in the severity of their depression scores, PROMs provide a critical patient-centred window into the benefit that inpatient treatment can have on those suffering with depression. These changes are consistent with those seen internationally and provide a baseline for understanding the treatment efficacy of an inpatient admission for the treatment of depression.
